# Phylogenetic analysis of mutational robustness based on codon usage supports that the standard genetic code does not prefer extreme environments

**DOI:** 10.1038/s41598-021-90440-y

**Published:** 2021-05-26

**Authors:** Ádám Radványi, Ádám Kun

**Affiliations:** 1 Department of Plant Systematics, Ecology and Theoretical Biology, Institute of Biology, Eötvös Loránd University, Budapest, Hungary; 2 Evolutionary Systems Research Group, Centre for Ecological Research, Institute of Evolution, Budapest, Hungary; 3 Parmenides Centre for the Conceptual Foundation of Science, Pullach, Germany; 4 MTA-ELTE Theoretical Biology and Evolutionary Ecology Research Group, Budapest, Hungary

**Keywords:** Translation, Molecular evolution, Phylogenetics, Information theory, Evolution

## Abstract

The mutational robustness of the genetic code is rarely discussed in the context of biological diversity, such as codon usage and related factors, often considered as independent of the actual organism’s proteome. Here we put the living beings back to picture and use distortion as a metric of mutational robustness. Distortion estimates the expected severities of non-synonymous mutations measuring it by amino acid physicochemical properties and weighting for codon usage. Using the biological variance of codon frequencies, we interpret the mutational robustness of the standard genetic code with regards to their corresponding environments and genomic compositions (GC-content). Employing phylogenetic analyses, we show that coding fidelity in physicochemical properties can deteriorate with codon usages adapted to extreme environments and these putative effects are not the artefacts of phylogenetic bias. High temperature environments select for codon usages with decreased mutational robustness of hydrophobic, volumetric, and isoelectric properties. Selection at high saline concentrations also leads to reduced fidelity in polar and isoelectric patterns. These show that the genetic code performs best with mesophilic codon usages, strengthening the view that LUCA or its ancestors preferred lower temperature environments. Taxonomic implications, such as rooting the tree of life, are also discussed.

## Introduction

The origin of translational apparatus and the genetic code is amongst the greatest conundrums of Life^1^. Its fundamental challenge is to uncover the constraints, historical accidents, and evolutionary driving forces that could have shaped the standard codon table. The current views propose the general mechanisms of (1) stereochemical affinity between codons and attributed amino acids (stereochemical theory^2,3^), (2) coevolution between the biosynthetic paths of amino acids and cognate codons (coevolution theory^4,5^), and (3) minimization of translation errors (adaptive, physicochemical or error minimization theory^6,7^) as possible explanations for the overall structure of the standard genetic code. Here, we focus and expand on the third one.

Although these existing hypotheses for the development of the genetic code are still hotly debated (including other theories, see other reviews^8,9^ for a recent overview of the field), the scientific community tends to agree on that the code is robust to mutations because its structure reduces the deleterious effects of translational errors. The error minimization theory proposes that the universal genetic code was shaped under selective forces that made the code at least partly optimized for fidelity in the physicochemical properties of mutated amino acids (or such aspects played a role during its development). With a few notable exceptions showing that the code can be locally improved with codon reassignments^10,11^, several studies have pointed out that the overwhelming portion of random alternative genetic code structures have inferior error capacities, leading to the argument of “optimality” of the standard genetic code^6,7,12^. The basis for these comparisons is the average fitness cost of replacing one amino acid with another due to mutation, measured on a scale of some physicochemical property (e.g. hydrophobicity or polarity).

Unfortunately, this is only one piece of the puzzle, because the majority of analyses operate under the implicit assumption that codons occur in a uniform distribution, which fails to address the possible variance in codon usage and its effect on robustness^13,14^. In contrast to this, a large body of studies has shown that adaptions to special environments and lifestyles, such as high salinity or extremely high temperatures, select for characteristic amino acid and codon compositions due to their special physicochemical requirements with regards to protein structure^15–25^. Also, there is an obvious correspondence between codon usage frequencies and GC-content^21,26,27^. The latter is associated with specific environments and lifestyles^28,29^ and mutational bias^30–32^.

Being aware of these facts, one can narrow the question further: Supposing that the universal genetic code is optimized for mutational robustness, in what condition does it have maximal efficiency? We could argue that it was the most likely environment to witness the final mappings between amino acids and their respective codons.

The information theoretic measure of distortion^33,34^ is a suitable concept to study the structure and “environmental behaviour” of the standard genetic code. In this framework, distortion is the expected average effect of mutations on the level of amino acid physicochemical properties, given a distribution of codon usage. It contains the same essentials as previous measures of code performance: (1) the estimate for the cost of faulty translation (one amino acid replaced by another), usually based on some physicochemical trait (e.g. hydrophobicity), and (2) the estimated probability of such translation errors being the result of a code in question. However, unlike conventional error definitions, distortion builds in a third term (3) by weighting for codon usage. Then, supplemented with a simple “background mutation model” (see “Methods” section), one can use distortion to compare the mutational robustness of codon usage profiles associated with different environments.

A previous study (Radványi and Kun, submitted) has suggested that extremophile codon usages might lead to diminished mutational robustness compared to that of mesophiles. Consequently, in thermophiles, the average effect of mutations, that is the distortion in physicochemical properties on the level of amino acids, was estimated to be greater than in mesophiles. Similar were the implications with regards to the GC-content of the coding region, implying a universal AT-bias. In that analysis, phylogenetic bias was not accounted for. Here we show that our result remains robust to taxon sampling.

## Methods

### Data

For codon usage profiles, we used the Uniprot Reference Proteome database within UniProtKB^35^. A Python script was used on the corresponding mRNAs to calculate nucleobase and codon distributions along with the distortion measures for each organism. This data was cross-referenced with optimal environmental conditions via NCBI Taxonomy ID-s^36^. The environmental data for optimal growth temperature, pH, and salt concentration were obtained from the BacDive database^37^. S16 rRNAs were retrieved from the 16S RefSeq Nucleotide^38^ sequence records. Sequences were aligned using MUSCLE^39^. The final dataset contained 64 taxa (8 archaeal and 56 bacterial), representative of the molecular diversity in each domain.

### Phylogenetic tree construction

We used Beast v1.10.4^40^ for a Bayesian analysis. The S16 rRNA phylogenetic tree was built from the 64-sequence alignment with a GTR^41^ model, a gamma law with eight categories and an estimated proportion of invariant sites, using default priors, and an uncorrelated relaxed clock. Chains were run for 50,000,000 generations and samples were collected in each 1000 generations. The analysis in Tracer^42^ demonstrated a good mixing of the chains. A burn-in of 5,000,000 samples was discarded, and a maximum clade credibility tree was computed from the remaining samples (Supplementary Data S1 online).

### Distortion as a measure of mutational robustness

In order to provide a practical measure for the error-rate of mistranslation and point-mutations, the information theoretic concept of distortion (Eq. 1)^33,34^ is used to estimate the average effect (cost per symbol) of mutations given a source distribution of codons and the uncertainty of the code resulting from noise, i.e. the probability of codon *c*_*i*_ mutating into *c*_*j*_ (see next section about background mutation model). Another important element of distortion is the distortion matrix, which reflects the underlying genetic code and corresponding physicochemical properties. This distortion matrix is essentially identical with matrices widely used in other studies, summarizing the errors of one amino acid mutating into another^6,7^. Distortion matrix with elements *d(aa*_*i*_*,aa*_*j*_*)* specifies the distortion associated with mistaking the encoded symbol *aa*_*i*_ (amino acid) in the source (*X*) and reproducing it as *aa*_*j*_ in the reproduced copy (*Y*). We define $d\left({aa}_{i},{aa}_{j}\right)=0$ if ${aa}_{i}={aa}_{j}$, that is, *c*_*i*_ and *c*_*j*_ codes for the same amino acid.

1 $$D=\sum_{i,j}{P(c}_{i})\times P\left(Y={c}_{j}|X={c}_{i}\right)\times d({aa}_{i},{aa}_{j})$$

Distinct distortion matrices were defined to provide different physicochemical measures of robustness. We decided to use properties made available by Haig and Hurst^7^. These include polar requirement, hydropathy, molecular volume and isoelectric point, yielding four different measures of code performance, denoted as *D*_Hyd_*, D*_Pol_*, D*_Vol_*, D*_pI_ (Supplementary Data S2 online).

### Background mutation model

The conditional probabilities *P(Y* = *c*_*j*_* | X* = *c*_*i*_*)* are the result of random mutations appearing in the genome and describe the chance of codon *c*_*i*_ mutating into codon *c*_*j*_. In order to approximate these probabilities, a simple background mutation model is required describing the generalized mechanism for spontaneous DNA mutations. However, we must estimate the raw, a priori performance of the genetic code without natural selection introducing additional bias. We introduce a simplified model of random amino acid mutations (Eqs.2–4), which essentially invokes Kimura’s two parameter model^43^. Here, *κ* denotes the transition/transversion rate ratio, otherwise known as ti/tv ratio; *p*_*ti*_ is the probability of transition, *p*_*tv*_ is the probability of transversion, and *µ* is the mutation rate. The inherent structure of the genetic code defines the probability of which codon *i* mutates into codon *j* given that a transition or transversion occurs; these are denoted by terms *P(c*_*i*_* → c*_*j*_* | ti)* and *P(c*_*i*_* → c*_*j*_* | tv)*, respectively.

2 $$\upkappa =\frac{{p}_{ti}}{{p}_{tv}}$$

3 $$P\left(Y={c}_{i}|X={c}_{j}\right)=\mu \times \left[\frac{\upkappa }{(1+\upkappa )}\times P\left({c}_{i}\to {c}_{j}|ti\right)+\frac{1}{(1+\upkappa )}\times P\left({c}_{i}\to {c}_{j}|tv\right)\right]$$

4 $$P\left(Y={c}_{i}|X={c}_{i}\right)=1-\mu$$

Expected proteomic distortions were then calculated for each taxon’s codon composition. Since our goal is a comparative analysis between taxa, the effect of *µ* is unimportant. Our preliminary work showed that although ti/tv ratio has a quantitative impact on distortion, the qualitative outcomes remain robust (Radványi and Kun, submitted). Our calculations concerning the distortion values were restricted to *κ* = 2.5 (roughly 71% of mutations are transitions), approximating ratios encountered by studies of genome-wide and intronic sequences^30,44^. Such regions ought to represent a more relaxed state of selection against mutations, hence providing a closer estimate of the background ratio of ti/tv prior to selection.

### Comparative analysis

To correct for non‐independence because of common ancestry of species, we performed Phylogenetic Generalized Least Squares (PGLS) models on the combined data. The analyses were carried out with the *ape*^45^, *phytools*^46^, *caper*^47^ and *geiger*^48^ packages in RStudio v3.5^49^. Response variables *D*_Hyd_*, D*_Pol_*, D*_Vol_ and *D*_pI_ were modelled separately using the available environmental variables as predictors. The genomic content of guanine and cytosine (GC-content) was also used as predictor since preliminary studies have shown its predominant effect on the codon and amino acid composition of proteomes^21,26,27^. Model assumptions were checked; no violations were apparent. Based on Akaike information criterion (AIC) values, we apply a Brownian model; further branch length transformations and correlation structures did not result in generally better fits.

## Results

**Figure 1:**
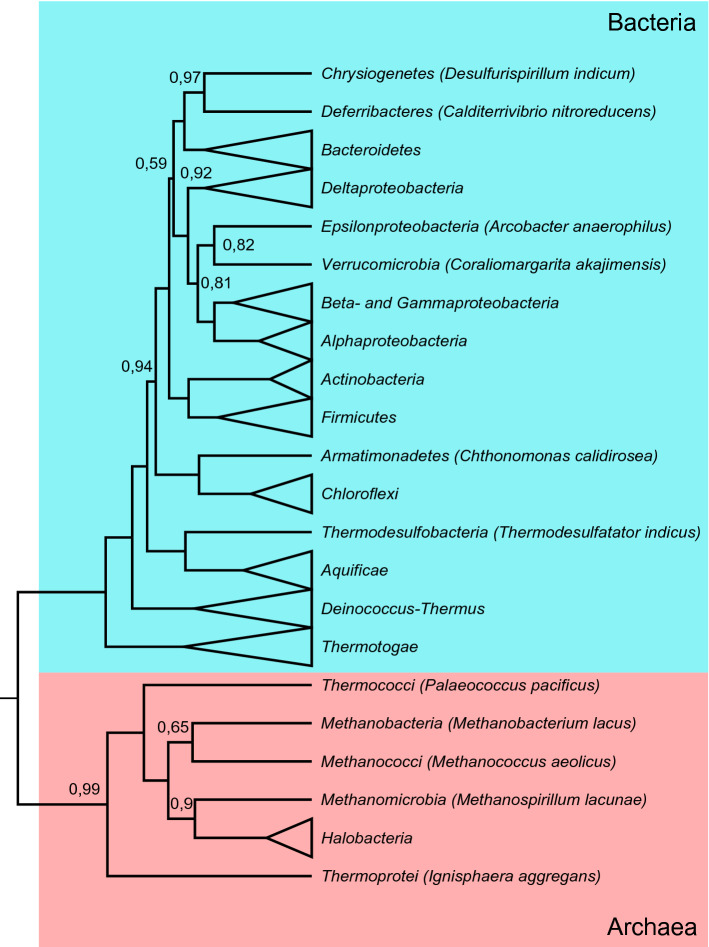
The large-scale hierarchy of the Bayesian phylogenetic tree based on S16 rRNAs sequences. The Bayesian tree was constructed using Beast v1.10.4^40^. The numbers at each node represent posterior probability values (only values < 1.00 are shown). Collapsed nodes contain multiple species (see Supplementary Data S1 online for more details).

**Figure 2:**
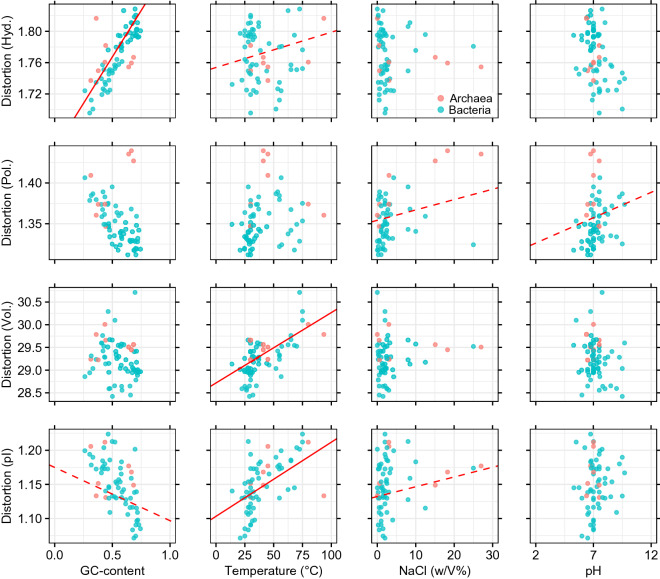
The effect of GC-content and environmental variables (temperature, salt concentration and pH) on the expected distortion measures (*κ* = 2.5) of different amino acid physicochemical properties. The effects were estimated by PGLS models using the inferred phylogenetic relationships shown on Fig. 1. Partial regression lines show significant trends (continuous line: *p* < 0.001, dashed line: *p* < 0.05). In each case, the remaining independent variables were set as [GC = 0.5; T = 30 °C; cc_NaCl_ = 2.5 w/V%; pH = 7].

In all four cases of physicochemical distortions, the PGLS resulted in significant models. In the obtained phylogenetic tree, deeper phylogenetic topologies are well supported by posterior values (Fig. 1). The results of the PGLS regressions are shown on Fig. 2, including partial regression lines. The standardized partial coefficients (where variables were Z-transformed prior to analysis) can be found as Supplementary Table S1 online. The highest proportion of explained variances is found in the case of distortion of hydrophobic properties (*R*^2^ = 0.633; *F*_4,59_ = 25.492; *p* = 2.726 × 10^−12^). The second highest proportion is explained for the distortion calculated for isoelectric points (*R*^2^ = 0.397; *F*_4,59_ = 9.689; *p* = 4.311 × 10^−6^), followed by that of the volumetric distortion (*R*^2^ = 0.361; F_4,59_ = 8.314; *p* = 2.180 × 10^−5^). The least amount of variance was found in the case of distortion in polar requirements (*R*^2^ = 0.249; *F*_4,59_ = 4.880; *p* = 1.816 × 10^−3^).

### The effect of GC-content

GC-content has a significant positive effect on hydrophobic distortion (*β* = 0.237; *t* = 9.464; *p* = 1.945 × 10^−13^). A less dominant, but significant negative effect is encountered in the distortion of isoelectric properties (*β* =  − 0.078; *t* =  − 2.147; *p* = 0.036). In other words, increasing the GC-content of the coding region results in lowered accuracy for hydrophobic traits, but the maintenance of molecular patterns related to isoelectric points becomes easier.

### Environmental effects

The effect of optimal growth temperature was positive and significant on hydrophobic (*β* = 4.510 × 10^−4^; *t* = 2.690; *p* = 0.009), volumetric (*β* = 0.015; *t* = 5.433; *p* = 1.100 × 10^−6^) and isoelectric distortion (*β* = 0.001; *t* = 4.467; *p* = 3.645 × 10^−5^); its effect on distortion in polar requirement was not significant. As for optimal NaCl concentration, significant positive effects were encountered with regards to distortions in polar requirement (*β* = 0.001; *t* = 2.479; *p* = 0.016) and isoelectric point (*β* = 0.001; *t* = 2.491; *p* = 0.016).

These effects translate to a general decrease in the expected physicochemical fidelity both in thermophiles and halophiles. In other words, such extremophilic codon usages could decrease the chance of preserving the respective physicochemical patterns with an occurring mutation, diminishing their mutational robustness.

The effect of ambient pH remains less conclusive. Although there is a significant positive effect on distortion in polar requirements (*β* = 0.006; *t* = 2.206; *p* = 0.031), other properties are not shown to be significantly influenced. Evidence of substantial selection on proteins in extreme acidophiles or alkaliphiles is sparse. This may be attributed to the relative invariance of intracellular pH regardless of the ambient environment^50^. We note, however, that our sample provides only a narrow pH range, and bias in codon usage have been recently noted^16^.

### Model robustness to ti/tv-ratio

To verify the robustness of these effects, we extended our analysis to a wider range of ti/tv-ratios (*κ* = 2.5–10; Supplementary Figure S1 online). The quantitative coefficients of the tested factors change asymptotically and remain significant. The signs of the significant coefficients do not change. The only exception is the GC-content where its negative impact on isoelectric properties becomes non-significant (*κ* > 2.5); therefore, only its positive effect on hydrophobic distortion is confirmed. Thus, we may conclude that the effects of environmental selection are robust on a broader range of ti/tv-ratios, and our interpretations concerning the impact of environmental selection, especially the distortive effect at high temperature, remains valid.

## Discussion

The optimality of the genetic code should be discussed in the context of different gradients, such as environmental selection or GC-content. Their possible repercussions on codon distribution will fundamentally impact the expected errors made by the code. In order to study this question, we have applied a previously developed minimalistic background mutation model for the generalised mechanism of emerging point-mutations. Then, we calculated distortions for codon distributions encountered in different taxa with known environmental requirements. Distortion measures were based on different physicochemical properties: hydropathy, polar requirement, volume, and isoelectric point.

Next, in order to account for phylogenetic non-independence, we performed four distinct Phylogenetic Generalized Least Squares (PGLS) regressions on these physicochemical distortion measures using a 16S rRNA tree (Fig. 1). One of the putative predictors was the GC-composition of the coding region of the genome, based on a large number of studies supporting its predominant effect on the amino acid composition of proteomes^26,51^. The other group of predictors included environmental variables, which can select for characteristic codon or amino acid compositions of proteomes: temperature, salt concentration, and pH optima.

The key insight provided in this study is that adaptations to certain extreme environments and GC-bias seem to have drastic effects on the physicochemical fidelity of translation and the severity of incidental mutations, and these putative effects are not the artefacts of phylogenetic bias.

While we have included only a limited number of taxa based on the availability of their proteomes and the environment they live in, they can be considered representative. Any phylogeny is constrained by what we know at the moment about the diversity of life on Earth. Current diversity is only a subset of the diversity that ever existed (which we need to keep in mind when we want to infer past events based on characteristics of current species), and metagenomics has repeatedly demonstrated that we know only a fraction of the current diversity. Metagenome studies discovered a previously unknown diversity of microbes^52,53^. Quite some of the microbial dark matter^54^ were first assigned to novel clades distinct from the established great groups of Bacteria and Archaea. Nowadays it seems, that the truly novel clades are fewer^54,55^. A recent catalogue of microbes, incorporating more than 50 thousand new metagenome-assembled genomes, concluded that the majority of deep-branching lineages (lineages that would be represented on the level of phylum) are represented by current genome sequences^56^; the Candidate Phyla Radiation^57^ could be merged into one monophyletic phylum^58^. Consequently, even a limited sample of taxa from all great branches of microbes can be considered representative.

### Substitution biases might be caused by aversions of certain physicochemical distortions

We have shown that high GC-content is expected to increase hydropathic distortion. Only this effect of nucleotide composition is robust to ti/tv-ratio, which would predict an AT-bias for the highest fidelity in hydrophobic attributes, pointing towards a general substitution trend that resembles preliminary observations of AT-biased substitution patterns^30–32^. Hydrophobic patterns are regarded as a primary force of protein folding^59–62^, further supported by the fact that secondary structures can be described and predicted along these properties^63^. Therefore, A/T-mutations should be more likely to fix due to their lower risk of jeopardizing the structure.

### Environmental selection influences the mutational robustness of the genetic code

With regards to environmental gradients, we show that selection in halophiles and thermophiles results in codon usage profiles generally worse for maintaining physicochemical patterns. Despite the observed adjustments against such mutations^16^ the conservation of the required physicochemical properties tends to be more unreliable and the average effects of incidental mutations are expected to be more severe and deleterious. Here, we have demonstrated reduced fidelity in hydrophobic, volumetric, and isoelectric patterns of thermophiles, as well as the vulnerability of halophiles to mutations disturbing polar and isoelectric arrangements in proteins. This is a possible explanation of why these extremophiles possess remarkably low mutation and substitution rates^64–68^: it is a straightforward result of avoiding harsh fitness costs, especially if we consider higher importance of hydrophobic interactions and salt bridges in thermophilic proteins^18,69^, as well as the central role of polar properties in halophiles^17,70^.

We conclude that such low mutation rates and strong selection patterns encountered in extremophiles are caused by the inefficiency of the genetic code, as it seems especially ill-suited for extremophile codon usages with regards to mutational robustness, which also means that evolvability diminishes with the employment of standard genetic code, casting doubts on what role extreme environments could have played at development of the codon mapping.

### Implications of the mesophile optimality of the genetic code and codon usage

Along with our estimated phylogeny, the majority of influential phylogenies have also provided an intuitive evidence of an extremophile LUCA, by placing thermophiles as the most basal groups^71–74^. This observation has long facilitated the somewhat overreaching logic that the cradle of life, including the development of the genetic code, was always associated with “infernal” environments of the Hadean Earth. At the same time, the genetic code is usually considered as a near-optimal, robust mapping that is able to partially maximize the fidelity of translation, since the majority, but not all^10,11^, of alternative codes falls short of such error capacity^6,7^.

Having these facts put together, our study implies a contradiction between these two notions: The fidelity of the genetic code is expected to decrease with higher temperatures. This not only collides with earlier reports supporting the extremophilic nature of the code^75–77^, but also points out that claims between its error-minimization and thermophilic origin seem non-compatible: if *“(and oh what a big if)”*^78^ the genetic code evolved in order to be optimal for physicochemical properties, then it is more likely to finish its emergence among milder conditions.

A mesophile optimality of the genetic code could be still compatible with phylogenies placing thermophiles at basal locations near LUCA. The evolution of the genetic code preceded LUCA. A well-thought-out rooting of the tree of life puts the bacterial clade *Chloroflexi* closest to the root^55^, and it has thermophilic members. *Chloroflexi* are photosynthetic bacteria, meaning that LUCA was an autotroph. But, the first cell was, by necessity, heterotrophic, i.e. dependent on the environment for organic building blocks; the fully fledged photosynthesis can evolve only later. This means that inferences about LUCA does not help us understand the environment in which the first cell or the organism inventing the standard genetic code thrived.

But there is no need to accept a thermophilic LUCA. Both rRNA and protein sequences indicate that hyperthermophilic features of Bacteria and Archaea are parallel adaptations, while their ancestors could have been mesophilic or only slightly thermophilic^79–81^. Furthermore, the idea of a thermophile LUCA comes from accepting the root of the universal tree of life to lay between Bacteria and Archaea. Cavalier-Smith has argued for quite some time, that the root lies within Gram negative bacteria, and Archaea and Eukaryotes (compromising the clade Neomura) are derived from Gram positive bacteria^82,83^. Archaea are the exemplars of extremophiles, but if their extremophilic characteristic is derived^84^, then there is no need for LUCA to be an extremophile. Indeed, among the *Chloroflexi*, there are mesophilic members, so even that does not contradict the proposition. Our own results presented here also strengthen the view that LUCA was a mesophile^85^.

### Outlook

Our work supports the expanded view on the optimality of the genetic code by involving codon usage^13,14^. The “mesophilic genetic code” hypothesis demands further research. Psychrophiles and organisms preferring extreme pH conditions could not be sufficiently represented in our current analysis, and albeit linear responses had a good fit in our case, increasing the environmental ranges should lead to more accurate estimations of translational preference. It must be also emphasized that our interpretation of codon usage analysis remains conditional on the assumption of error minimization. It does not weaken the case of other dominant theories of the evolution of the genetic code. The evolution of genetic code is likely to be a result of multiple driving forces and cannot be understood solely by natural selection increasing mutational robustness^86^.

Relying on simple codon usage data have disadvantages. Thermophiles and halophiles possess elevated rates of horizontal gene transfer^87–89^. The codon bias of recently acquired, not completely adapted genes originally hosted by non-extremophilic hosts can interfere with the analysis, thus a later focus on core genes is needed. Upcoming studies are also yet to address the effect of mRNA expression patterns. Here, the implicit assumption is that proteins are expressed on the same level in each proteome. However, difference in expression has clear evolutional implications^90^ that are also related to thermophilic properties^91^ and misfold chance^92^. We believe that these observations can be incorporated into theory.

On the other hand, our result should not be taken as a direct confirmation of the universal genetic code being an inefficient mapping at extreme conditions. To assess that point better, it would demand us to gather data about the robustness of alternative codes and the proportion of variants where codon usage response to extreme environments can increase fidelity.

There is another point where biological codon usage data fails to elaborate. Simply extending the measure of distortion to the domain of alternative codes poses a paramount challenge. The currently known codon usage profiles cover only the standard genetic code (and alternative genetic code variants to some extent^14^). As codon frequencies and their environmental response already carry the inherent effect of the standard genetic code, their variance cannot be directly applied to randomized codes. This warrants further investigation into the physicochemical requirements of extreme habitat conditions as the likely causes of characteristic amino acid compositions that influence codon distribution.

## Supplementary Information

Supplementary Information.

Supplementary Data 1.

Supplementary Data 2.

## Data Availability

All data generated or analysed during this study are included in this published article (and its Supplementary Information files)**.**
