# Supplementary material for: Phylogenetic analysis of mutational robustness based on codon usage supports that the standard genetic code does not prefer extreme environments
Source: Sci Rep. 2021 May 26;11:10963. doi: 10.1038/s41598-021-90440-y (PMC8154912; doi:10.1038/s41598-021-90440-y)
Supplement: Supplementary file 1 — Supplementary Information. [file 41598_2021_90440_MOESM1_ESM.docx]

Phylogenetic analysis of mutational robustness based on codon usage supports that the standard genetic code does not prefer extreme environments

Ádám Radványi^1*^ and Ádám Kun^2,3,4^

^1^ Department of Plant Systematics, Ecology and Theoretical Biology, Institute of Biology, Eötvös Loránd University, Budapest, Hungary

^2^ Evolutionary Systems Research Group, Institute of Evolution, Centre for Ecological Research, Tihany, Hungary

^3^ Parmenides Centre for the Conceptual Foundation of Science, Pullach, Germany

^4^ MTA-ELTE Theoretical Biology and Evolutionary Ecology Research Group, Budapest, Hungary

e-mail: [adamradvanyi117@gmail.com](mailto:adamradvanyi117@gmail.com)

# Supplementary Information


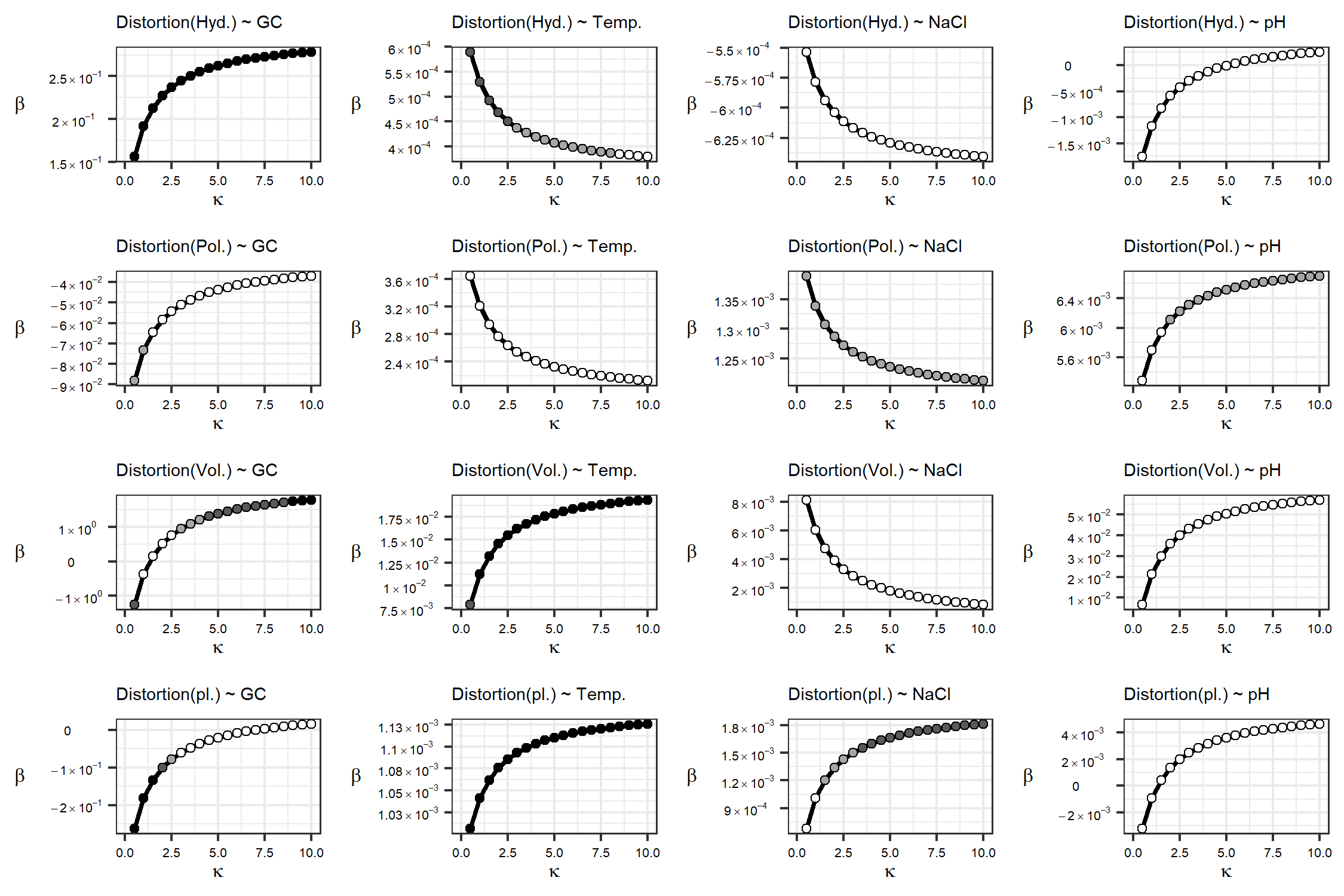


**Figure S1.** The effect of ti/tv-ratio (κ) on the coefficients of tested variables (β). Different ti/tv-ratios are used in the background mutation model to measure the change in coefficients of the PGLS analyses. The significance level of coefficients is marked by shade (black: p<0.001, dark grey: p<0.01, light grey: p<0.05 white: p>0.05).

**Table S1.** PGLS results (distortions calculated at κ=2.5) for variables standardized prior to analysis.

| Dependent var. (standardized) | D_Hyd_ R^2^=0.633  (F_4,59_=25.492; p=2.726×10^-12^) | | | | D_Pol_ R^2^=0.249  (F_4,59_=4.880; p=1.816×10^-3^) | | | | D_Vol_ R^2^=0.361  (F_4,59_=8.314; p=2.180×10^-5^) | | | | D_pI_ R^2^=0.397  (F_4,59_=9.689; p=4.311×10^-6^) | | | |
| --- | --- | --- | --- | --- | --- | --- | --- | --- | --- | --- | --- | --- | --- | --- | --- | --- |
| Parameter | *β* | SE | *t* | sig. | *β* | SE | *t* | sig. | *β* | SE | *t* | sig. | *β* | SE | *t* | sig. |
| (Intercept) | 0.382 | 0.390 | 0.979 | 0.332 | 0.214 | 0.562 | 0.380 | 0.705 | 0.331 | 0.506 | 0.654 | 0.515 | -0.095 | 0.500 | -0.191 | 0.850 |
| GC-percent | 0.924 | 0.098 | 9.464 | 0.000 | -0.235 | 0.141 | -1.671 | 0.100 | 0.225 | 0.126 | 1.782 | 0.080 | -0.268 | 0.125 | -2.148 | 0.036 |
| Temperature | 0.232 | 0.086 | 2.690 | 0.009 | 0.151 | 0.124 | 1.216 | 0.229 | 0.607 | 0.112 | 5.434 | 0.000 | 0.492 | 0.110 | 4.467 | 0.000 |
| NaCl conc. | -0.099 | 0.064 | -1.543 | 0.128 | 0.230 | 0.093 | 2.478 | 0.016 | 0.041 | 0.083 | 0.492 | 0.624 | 0.205 | 0.082 | 2.492 | 0.016 |
| pH | -0.011 | 0.059 | -0.195 | 0.846 | 0.187 | 0.085 | 2.206 | 0.031 | 0.083 | 0.076 | 1.083 | 0.283 | 0.048 | 0.075 | 0.633 | 0.529 |

**Data S1.** The inferred maximum clade credibility tree used in PGLS analyses (format: JSON). Branch lengths and posterior values are included.

**Data S2.** Dataset used in PGLS regressions. Includes Uniprot Proteome references and NCBI Taxonomy IDs, optimal growth environment data, nucleobase and codon frequencies, calculated distortion values and additional taxonomic information.
